# Enhancing cooking and eating quality of semi-dried brown rice noodles through Lactobacillus fermentation and moderate lysine addition

**DOI:** 10.1016/j.fochx.2025.102327

**Published:** 2025-03-06

**Authors:** Lijuan Luo, Gangping Xiong, Qing Wang, Xiongzi Xiang, Zhao Long, Zhengyu Huang, Yuqin Ding, Chun Liu

**Affiliations:** a National Engineering Research Center for Rice and By-product Deep Processing, School of Food Science and Engineering, Central South University of Forestry and Technology, Changsha 410004, China; b Sericultural & Agri-Food Research Institute Guangdong Academy of Agricultural Sciences, Key Laboratory of Functional Foods, Ministry of Agriculture and Rural Affairs, Guangdong Key Laboratory of Agricultural Products Processing, Guangzhou 510610, China

**Keywords:** Fermented Brown Rice noodles, Lysine addition, Sensory evaluation, Cooking quality, Texture

## Abstract

Various concentrations of lysine were added to *Lactobacillus fermentum*-fermented brown rice flour to examine their effects on the sensory evaluation, color, pH, cooking quality, and texture of the resulting noodles. The potential mechanisms were explored through analyses of water distribution, thermodynamic properties, crystallinity, and microstructural observations using low-field nuclear magnetic resonance (LF-NMR), differential scanning calorimetry (DSC), X-ray diffraction (XRD), and scanning electron microscopy (SEM). Low concentrations of lysine (1 % and 3 %) effectively mitigated sourness while maintaining a color similar to natural brown rice noodles, with minimal structural damage and good cooking quality. The 3 % lysine group received the highest sensory scores (73.3 ± 5.4), likely due to moderate lysine-mediated starch network formation and improved water retention. While, a high lysine concentration (5 %) resulted in an undesirable reddish-brown color, poor texture and cooking quality. This is likely due to the formation of rigid lysine-mediated starch structures, which made the noodles brittle.

## Highlights

• Lysine addition mitigates sourness in Lactobacillus-fermented brown rice noodles.
• 1 % and 3 % lysine preserve noodle color and texture, enhancing cooking quality.
• High lysine levels (5 %) result in undesirable color and poor noodle texture.
• Lysine enhances inter-starch bonds, boosting water retention and gel properties.

## 1 Introduction

Brown rice is a whole grain rich in essential nutrients such as fiber, vitamins, minerals, and bioactive compounds, offering health benefits like improved cardiovascular health and digestive wellness (Ravichanthiran et al., 2018). Despite its nutritional advantages, brown rice's coarser texture and longer cooking time limit its widespread use (Chao et al., 2021). To enhance its appeal, brown rice-based products like noodles have been developed, with semi-dry rice noodles gaining popularity for their convenience and closer texture to fresh noodles (Xiao et al., 2022).

Fermentation with lactic acid bacteria (LAB) has been shown to improve rice noodle quality by enhancing texture (Geng et al., 2019; Zhu et al., 2019). However, a major challenge with LAB fermentation is the production of excess organic acids, which can impart an undesirable sour taste. While various methods can mitigate sourness, including processing techniques or ingredient modifications (Mishra et al., 2017). Novel methods like high hydrostatic pressure, ionizing radiation, and ultrasound can reduce sourness while maintaining product quality (Olatunde et al., 2023). Adjusting factors such as salt levels, temperature, and enzyme activity can also mitigate sourness by influencing flavor development (McFeeters, 2004). The addition of amino acids like l-lysine has also shown potential in improving sensory qualities. l-lysine has been reported to reduce sourness and enhance flavor in food products. For instance, Zhang et al., 2024 demonstrated that incorporating 0.3 % l-lysine in reduced‑sodium frankfurters reduced cooking loss, improved water content, and balanced the sensory profile by reducing sourness, astringency, and bitterness while enhancing umami and richness. Similarly, Zhou et al., 2014 found that 0.8 % l-lysine improved water-holding capacity, pH, texture, and cooking properties of pork sausages. Furthermore, Lu et al., 2012 showed that adding lysine to maize starch influenced its physical properties, such as increasing peak viscosity and solubility while decreasing pasting temperature. While there is evidence suggesting that lysine could mitigate the sour taste from fermentation and effect the overall quality of starch-based noodles, these effects may vary depending on the concentration of lysine added (Albert et al., 2017).

Despite the promising findings on the individual effects of lysine and LAB fermentation, there is limited research on their combined impact on the quality of rice noodles. Most studies focus on either lysine addition or fermentation alone, leaving a gap in understanding how these two factors interact in the context of rice noodles made from fermented flour. Moreover, while the effects of lysine on wheat-based and white rice noodles are documented, its impact on brown rice noodles, particularly those made from fermented brown rice, remains underexplored.

This study aims to investigate the effects of adding different concentrations of lysine (1 %, 3 %, and 5 % *w*/w) on the quality of rice noodles made from LAB-fermented brown rice flour. The research will not only identify the optimal lysine concentration that enhances the noodles' acceptability but also explore the underlying mechanisms by which lysine influences these properties. The study will focus on key quality parameters such as sensory attributes, pH value, color differences, cooking characteristics, and texture properties. Additionally, it will examine mechanistic indicators such as water distribution, cross-sectional morphology, crystallinity, and thermodynamic properties to provide a comprehensive understanding of how lysine interacts with the fermentation process to modify the quality of brown rice noodles.

## 2 Materials and methods

### 2.1 Materials

The early indica rice variety ‘Zhenzhusanhao’ was selected for this study. The rice, having been stored for one to two years, was husked and impurities were removed. The cleaned rice was then ground using a grinder and sieved through a 100 mesh screen to obtain brown rice powder. The brown rice powder was subsequently stored at 4 °C until further use. Food-grade lysine was purchased from Henan Wanbang Chemical Technology Co., LTD. All other chemicals used in this study were of analytical grade.

### 2.2 Methods

#### 2.2.1 Preparation of fermented brown rice powder

Brown rice powder was fermented with a 10 % inoculum of *Lactobacillus fermentum* (2.5 × 10^8^ CFU/mL) at a 1:0.9 feed-to-water ratio (*w*/w) for 72 h at 30 °C. Humidity was controlled at 80 %, and aeration was provided by gentle stirring. Post-fermentation, the samples were freeze-dried for subsequent analyses. The *Lactobacillus fermentum* strain (*L.fermentum-L6*) was isolated from Jinjian Cereals Industry fermentation broth as described in our previous work (Luo et al., 2024). The dominant lactic acid bacteria strain was identified using the streak plate method on MRS broth medium, incubated at 30 °C for 24 h. After 16S rRNA gene identification, the strain was confirmed as *Lactobacillus fermentum*.

#### 2.2.2 Preparation of semi-dry Brown Rice noodles enriched with lysine

The preparation method for semi-dry rice noodles followed our previously improved protocol (Luo et al., 2024). Brown rice flour was mixed with food-grade lysine at concentrations of 0 %, 1 %, 3 %, and 5 % (*w*/w) based on the dry weight of the flour. Distilled water, at 1.25 times the weight of the brown rice flour, was added to each mixture. The mixtures were then thoroughly stirred to ensure uniform distribution. The resultant dough was pre-gelatinized by heating in a water bath at 98 °C for 8 min with continuous stirring. The pre-gelatinized dough was kneaded into a cohesive mass, loaded into an manual extruder, and extruded into noodle shapes. The extruded noodles were spread evenly in a steaming tray and steamed at 100 °C for 3 min. Subsequently, the steamed noodles were cooled to room temperature and aged in a refrigerator at 4 °C for 4 h. After aging, the noodles were vacuum-packed in vacuum-sealed bags and sterilized in a water bath at 98 °C for 30 min. Following sterilization, the noodles were rapidly cooled in ice water for 5 min. Finally, the sterilized noodles were removed, stored in a cool and dry place, and subjected to various quality assessments. The unfermented brown rice noodles, fermented brown rice noodles, and fermented noodles with 1 %, 3 %, and 5 % lysine added were labeled as NBRN, FBRN, FBRN-1 L, FBRN-3 L, and FBRN-5 L, respectively.

#### 2.2.3 Sensory evaluation

The sensory properties of the brown rice noodles were evaluated following the standard method for sensory evaluation of edible rice noodles (LS/T 6137–2020) issued by the National Food and Strategic Reserves Administration of China. The evaluation criteria were slightly modified based this standard to better suit the characteristics of the brown rice noodles (Table S1). Twenty assessors (10 females and 10 males) were selected. Water was provided to cleanse their palates between samples. Samples, placed in plastic cups, were presented to the panelists in a randomized order, with evaluations completed within 10 min of sample preparation. Each panelist, in individual booths, evaluated one sample at a time for odor (0–15 points), appearance (0–25 points), texture (0–35 points), and taste (0–25 points). The sensory evaluation complied with the relevant laws and institutional guidelines. Since the study involved non-invasive testing with no personal data collection, ethical approval was not required. All participants gave 134 their informed consent, and their rights and privacy were fully protected throughout the process.

#### 2.2.4 pH value measurement

Based on the modified method of Xiao et al., 2023, the pH value of the rice noodles was determined using a pH meter (FE28; Mettler Toledo, Shanghai, China). A 10 g uniform rice noodle sample (accurate to 0.01 g) was weighed and crushed. Freshly boiled and cooled water was added to the sample to make up a volume of 100 mL. The mixture was shaken well and soaked for 30 min. After soaking, the mixture was filtered, and the pH value was measured using the pH meter.

#### 2.2.5 Chromaticity value measurement

Based on the modified method of Xiao et al., 2023, a hand-held colorimeter (CR-400; Konica Minolta Holdings, Inc., Japan) was used to measure the chromaticity value of the rice noodles. The samples were dried in an oven at 45 °C for 10 h, then crushed and sieved to obtain 40–80 mesh particles. These particles were stored in ziplock bags for later use. After preheating the colorimeter, the ziplock bag containing the sample was placed into the measuring port for measurement. The L*, a*, and b* values were recorded, and each measurement was repeated five times for accuracy.

#### 2.2.6 Cooking quality

(1) Broken Strips Rate

Three portions of rice noodles, each containing 20 pieces of approximately 20 cm in length, were placed in a beaker with 300 mL of boiling water and slightly boiled for 3 min. The noodles were then removed with a strainer, cooled in tap water for 1 min, and drained for 5 min. The noodles were separated into pieces shorter than 10 cm and those longer than 10 cm, and their weights were recorded. Each sample was tested in triplicate. The broken strips rate was calculated using the following formula:

Broken strips rate (%) = $\left(\frac{\text{Weight of rice noodles less than}\;10\mathrm{cm}\;\text{in length}}{\text{Total weight of rice noodles less than}\;10\mathrm{cm}\;\text{and more than}\;10\mathrm{cm}\;\text{in length}}\right)\times 100$

(2) Water Absorption Rate

Three portions of 20 pieces of rice noodles, each approximately 20 cm in length, were accurately weighed (to 0.01 g). The noodles were placed in a beaker with 300 mL of boiling water and slightly boiled for 3 min. The noodles were then removed with a strainer, cooled in tap water for 1 min, drained for 5 min, and weighed again. The water absorption rate was calculated as follows:

Water Absorption Rate (%) = $\left(\frac{\text{Weight of the rice noodles after cooking}}{\text{Initial weight of the rice noodles}}\right)\times 100\%$

(3) Cooking Loss Rate

The cooking loss rate was determined using a method adapted from Xiao et al., 2023. Three portions of 20 rice noodles, each approximately 20 cm in length, were accurately weighed and recorded as m0. Following the cooking procedure, the collected cooking and rinsing water were evaporated on an electric stove until most of the water was removed. The residue was then dried in a forced-air oven at 105 °C until reaching a constant weight, recorded as m1. The moisture content (c) of the rice noodle samples was measured by drying them in an oven at 105 °C. The cooking loss rate was calculated using the following formula:

$$\mathrm{Cooking Loss Rate}\;\left(\%\right)=\frac{m1}{m0\times \left(1-c\right)}$$

(4) Iodine Blue Value

The iodine blue value was determined based on the method of Xiao et al., 2023, with modifications. Briefly, 2.5 g of rice noodles were added to 50 mL of boiling distilled water and boiled for 3 min. The filtrate (10 mL) was mixed with 1 mL of 1 mol/L HCl and 1 mL of iodine reagent, then diluted to 50 mL with distilled water and shaken thoroughly. After standing for 10 min, the absorbance was measured at 620 nm. A blank was prepared by mixing 1 mL of 1 mol/L HCl and 1 mL of iodine reagent, and diluting to 50 mL with distilled water.

#### 2.2.7 Texture analysis

Based on the modified method of Xiao et al., 2023, the textural properties of the rice noodles were determined using a TPA Texture Analyzer (TA XT Plus, Stable Micro System Ltd., UK). Ten rice noodles, each approximately 20 cm in length, were cooked in boiling water for 3 min, rinsed with distilled water for 30 s, and then drained. The TPA mode was used with a p/36R probe, and the test parameters were set as follows: pre-test speed 1 mm/s, test speed 1 mm/s, post-test speed 1 mm/s, touching pressure 5 g, and compression ratio 75 %. Each sample was measured 10 times for accuracy and repeatability.

#### 2.2.8 Moisture distribution

Based on the modified method of Xiao et al., 2023, the moisture distribution in the rice noodles was determined using a low-field nuclear magnetic resonance (LF-NMR) analyzer (NMI20; Shanghai NewMai Electronic Technology Co., Ltd., China). The rice noodles were cut into small segments, each 3 cm in length. Ten segments were selected and placed in NMR tubes, which were sealed with plastic wrap to prevent water evaporation. The spin-spin relaxation time (T2) of the samples was determined using the Carr-Purcell-Meiboom-Gill (CPMG) pulse sequence. The sequence parameters were set as follows: spectral width (SW) 200 kHz, waiting time (TW) 2000 ms, number of scans (NS) 32, and number of echoes (NECH) 2000.

#### 2.2.9 Thermodynamic characteristics

Based on the modified method of Xiao et al., 2023, the thermodynamic properties of the rice noodles were characterized using a differential scanning calorimeter (DSC) (DSCQ2000; Waters, USA). The rice noodles were dried in an oven at 45 °C for 10 h and then passed through a 100-mesh sieve. Approximately 2.0 mg of the sieved sample was weighed into a PE liquid aluminum crucible. Then, 6 μL of deionized water was added to the crucible, which was capped, sealed, and equilibrated overnight. The test procedure involved heating the sample at a rate of 10 °C per minute over a temperature range of 20 °C to 120 °C. The onset temperature (To), peak temperature (Tp), endset temperature (Te), and enthalpy change (∆H) were recorded.

#### 2.2.10 Crystallinity

Based on the modified method of Xiao et al., 2023, the crystallinity of the rice noodles was determined using an X-ray diffractometer (XRD) (Rigaku Smart Lab SE; Rigaku, Japan). The rice noodles were dried in an oven at 45 °C for 10 h and then passed through a 200-mesh sieve. The XRD experimental parameters were as follows: Cu-Kα target, tube voltage of 30 kV, electric current of 10 mA, scanning interval of 2*θ* = 5–35°, step size of 0.02°, and scanning speed of 5°/min. The results were analyzed using MDI Jade software.

#### 2.2.11 Microstructure of Rice noodle cross-section

Based on the modified method of Xiao et al., 2023, the microstructure of the freeze-dried rice noodles was observed using scanning electron microscopy (SEM) (Tescan MIRA LMS; Tescan, Czech Republic). The rice noodles were cut into small segments of 5 cm in length, lyophilized in a vacuum freeze dryer, and then stored in a 10 mL centrifuge tube until measurement.

#### 2.2.12 Statistical analysis

Except where otherwise noted, all measurements were performed in triplicate. A one-way analysis of variance (ANOVA) and Duncan's multiple range test were used to test and compare means at a significance level of 5 %, utilizing IBM SPSS 19.0 software (SPSS Inc., Chicago, Illinois, USA).

## 3 Results and discussion

### 3.1 Impact of lysine addition on the sensory quality of fermented semi-dry Brown Rice noodles

**Fig. 1:**
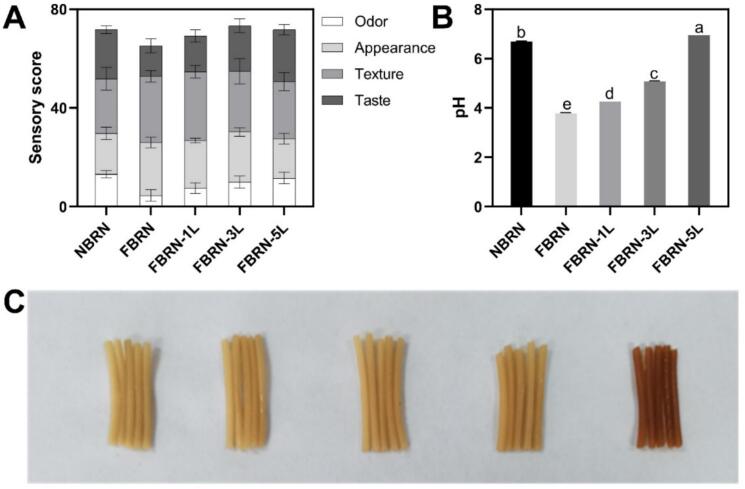
Effects of fermentation and lysine addition on the sensory scores (A) and pH values (B) of brown rice noodles. (C) Visual comparison of brown rice noodles (From left to right: NBRN, FBRN, FBRN-1 L, FBRN-3 L, FBRN-5 L). (For interpretation of the references to color in this figure legend, the reader is referred to the web version of this article.)

Fig. 1A and Table S2 presents the sensory quality of fermented semi-dry brown rice noodles with different concentrations of added lysine. Compared to non-fermented brown rice noodles (NBRN), the noodles prepared from fermented brown rice flours (FBRN) exhibited lower scores in odor and taste but significantly higher scores in appearance and texture. The introduction of lysine improved the odor and taste scores of FBRN. The addition of l-lysine to fermented rice noodles mitigated acidity, possibly because it buffers the organic acids and neutralizes the excess acid produced during fermentation, thereby improving the aroma and taste of the noodles. As the lysine concentration increased, the improvements in these sensory attributes were more pronounced. However, the addition of lysine either maintained or deteriorated the appearance and texture of FBRN, with more deterioration observed at higher lysine concentrations (5 %), although they remained comparable to NBRN. Among the test groups, FBRN-3 L achieved the highest sensory scores (73.3 ± 5.4), identifying it as the optimal balance. This group had notably higher scores in odor and taste while maintaining appearance and texture scores comparable to the pure fermentation group (FBRN).

### 3.2 Effect of lysine addition on the pH of fermented semi-dry Brown Rice noodles

Fig. 1B illustrates the effect of lysine addition on the pH of fermented semi-dry brown rice noodles. NBRN have a pH of 6.71. Fermentation significantly reduces the pH of the noodles to 3.79 (FBRN). However, the addition of lysine raises the pH progressively with increasing lysine concentrations. Specifically, the pH values are 4.27 for FBRN-1 L, 5.09 for FBRN-3 L, and 6.97 for FBRN-5 L, the latter even exceeding the pH of NBRN. These results demonstrate that lysine addition mitigates the acidity induced by fermentation, suggesting that lysine serves not only as a nutritional enhancement but also as a pH modulator in the fermented product. A similar phenomenon was observed by Zhou et al., 2014, who found that 0.8 % l-lysine improved the pH of pork sausages.

### 3.3 Impact of lysine addition on the color differences of fermented semi-dry Brown Rice noodles

Table S3 and Fig. 1C compare the color difference values and actual color of fermented semi-dry brown rice noodles with added lysine. The appearance quality of noodles directly impacts consumer acceptance. Compared to NBRN, FBRN exhibit higher brightness (L* value) and lower redness and yellowness (a* and b* values), likely due to lactic acid fermentation inhibiting Maillard browning by lowering pH and reducing reactant availability (Kathuria et al., 2023). However, lysine addition decreases the L* value and increases the a* and b* values, resulting in a darker color. At lower lysine concentrations (1 % and 3 %), the L*, a*, and b* values are similar to or better than those of NBRN, maintaining a comparable appearance and brightness (Fig. 1C). In contrast, higher lysine concentrations (5 %) significantly darken the color to reddish-brown, likely due to the excess lysine reacting with reducing sugars and promoting the Maillard reaction and caramelization under elevated pH conditions (Ajandouz et al., 2001; Treibmann et al., 2017). This observation is consistent with findings from Dai et al., 2023, who reported that the addition of lysine to a soy protein-wheat gluten mixture subjected to high-moisture extrusion also deepened the color of the extrudates. Therefore, careful consideration of lysine concentration is essential to balance nutritional enhancement and maintain desirable noodle color.

### 3.4 Effect of lysine addition on the cooking characteristics of fermented semi-dry brown rice noodles

**Fig. 2:**
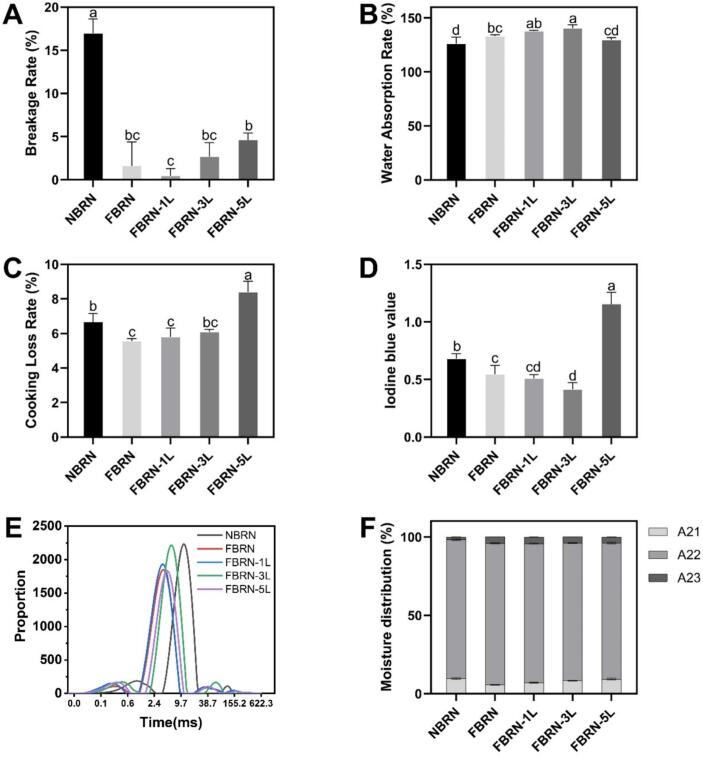
Effects of fermentation and lysine addition on the cooking quality of brown rice noodles. (A) Breakage rate, (B) Water absorption rate, (C) Cooking loss rate, (D) Iodine blue value. Effects of fermentation and lysine addition on the Spin-Spin relaxation time spectra (T2) (E) and moisture distribution (F) of brown rice noodles. (For interpretation of the references to color in this figure legend, the reader is referred to the web version of this article.)

From Fig. 2A-D, it can be seen that compared to NBRN, fermentation improved the cooking quality of brown rice noodles. The breakage rate in FBRN significantly reduced to 1.6 % from 17.0 % in NBRN, indicating enhanced structural integrity. Water absorption rate increased to 133.1 % from 126.2 %, suggesting better hydration. The cooking loss rate decreased to 5.6 % from 6.7 %, reflecting better retention of structural components during cooking. Additionally, the iodine blue value reduced to 0.5 from 0.7, indicating effective structural stability and minimal starch leakage.

With the addition of lysine, further variations in cooking quality were observed. The breakage rate, initially low in the FBRN-1 L (0.5 %), increased with higher lysine concentrations, peaking at 4.6 % in the FBRN-5 L. Water absorption showed an optimal increase at FBRN-3 L (140.6 %) but decreased at FBRN-5 L (129.5 %). Cooking loss was consistently low at FBRN-1 L and FBRN-3 L (5.8 % and 6.1 %, respectively) but significantly increased at FBRN-5 L (8.4 %). The iodine blue value, indicating starch leakage, was lowest at FBRN-3 L (0.4) and highest at FBRN-5 L (1.2). Overall, both FBRN-1 L and FBRN-3 L exhibited cooking qualities comparable to FBRN, with enhanced water absorption and minimized starch leakage, demonstrating better structural stability during cooking. However, FBRN-5 L showed diminished cooking qualities, such as higher breakage rates and compromised structural integrity. This degradation in quality may be due to excessive lysine promoting overly strong interactions among starch molecules, resulting in increased brittleness and reduced cooking quality. These findings underscore the necessity of optimizing lysine concentration to achieve the best balance of cooking quality in fermented semi-dry brown rice noodles.

### 3.5 Effect of lysine addition on the texture of fermented semi-dry Brown Rice noodles

From Table 1, the texture analysis revealed that fermentation improved noodle quality by decreasing hardness and enhancing springiness while maintaining cohesiveness. When lysine was added to the FBRN group, it led to an initial increase in hardness in FBRN-1 L but a decrease in FBRN-3 L and FBRN-5 L. Adhesiveness increased with lysine concentration, reaching the highest in FBRN-5 L. This increase in adhesiveness generally correlates with higher stickiness, which may be perceived as undesirable by consumers, affecting the mouthfeel and texture negatively. Springiness showed a mild reduction, reflecting slight effects on elasticity. Cohesiveness remained relatively stable across all groups. Gumminess initially increased in FBRN-1 L but decreased in FBRN-3 L and FBRN-5 L. Chewiness followed a similar pattern, increasing in FBRN-1 L and decreasing in FBRN-3 L and FBRN-5 L. Resilience was higher in FBRN-1 L and FBRN-3 L but lower in the FBRN-5 L. To sum up, FBRN-1 L and FBRN-3 L exhibit desirable textural properties such as moderate hardness, low stickiness, and high resilience. In contrast, FBRN-5 L exhibits poor textural properties, with low hardness, high stickiness, and low resilience. The possible reason is that low concentrations of lysine promote interactions between starch molecules, enhancing the network structure, which improves texture. However, at higher concentrations, excessive lysine can lead to overly strong interactions, creating a dense, rigid starch matrix that reduces chewiness and increases brittleness, resulting in a dense and more brittle texture.

**Table 1:** Effects of fermentation and lysine addition on the textural properties of semi-dry brown rice noodles.

Samples	Hardness/g	Adhesiveness (g.s)	Springiness/%	Cohesiveness	Gumminess	Chewiness	Resilience/%
NBRN	8694 ± 194a	−579 ± 30a	52.4 ± 6.1b	0.368 ± 0.016a	3197 ± 170ab	1671 ± 151b	16.1 ± 1.2ab
FBRN	7959 ± 103b	−568 ± 46a	62.8 ± 2.4a	0.383 ± 0.012a	3049 ± 54b	1913 ± 65a	15.0 ± 0.5b
FBRN-1 L	8480 ± 162a	−671 ± 165ab	57.2 ± 2.7ab	0.396 ± 0.010a	3355 ± 142a	1916 ± 75a	17.7 ± 1.4a
FBRN-3 L	6975 ± 120c	−540 ± 85a	54.3 ± 3.3b	0.391 ± 0.020a	2725 ± 103c	1476 ± 41c	17.3 ± 0.8a
FBRN-5 L	6349 ± 104d	−792 ± 31b	59.3 ± 1.7ab	0.393 ± 0.014a	2499 ± 126c	1483 ± 107c	15.3 ± 0.4b

Note: Values are presented as mean ± standard deviation. Different letters within the same column indicate significant differences. Adhesiveness values with a negative sign indicate the force needed to separate the sample from the probe. A more negative value suggests higher stickiness or increased adhesive force between the sample and the probe.

### 3.6 Effect of lysine addition on the water distribution of fermented semi-dry Brown Rice noodles

The water content, location, mobility, and interaction with other food matrix components significantly affect the stability and functional properties of starchy foods (Chen et al., 2017; Wang et al., 2016). Low-field nuclear magnetic resonance (LF-NMR) technology can analyze water distribution (strongly bound water, weakly bound water, and free water) and water migration in noodles. Fig. 2E shows the spin-spin relaxation time T2 spectra of fermented brown rice noodles, displaying three peaks corresponding to T21 (0.01–1 ms, bound water), T22 (1–10 ms, weakly bound water), and T23 (>10 ms, free water) (Wang et al., 2022). A larger T2 value indicates greater water mobility. Compared to NBRN, the T2 curve for FBRN shifted significantly to the left, indicating reduced water mobility. With the addition of lysine, no significant change in relaxation time was observed for FBRN-1 L. FBRN-3 L shifted rightward, approaching the relaxation time of NBRN, while FBRN-5 L shifted left again. Fig. 2F and Table S4 shows the peak areas A21, A22, and A23, representing the content of bound water, weakly bound water, and free water, respectively. NBRN primarily consist of weakly bound water. Fermentation reduced the content of bound water and increased the weakly bound and free water content. With increasing lysine content, bound water content gradually increased, while weakly bound and free water content decreased, demonstrating the addition of lysine can improve the water-retaining property in starch. This improvement is due to lysine promoting specific interactions within the starch molecules, leading to a rearrangement that increases tightly bound water content, thereby enhancing noodle gel properties and water-holding capacity. Similarly, Huang et al. also found that the addition of lysine in plant-protein high-moisture extrudates elevates tightly bound water content by stimulating protein aggregation and forming network structures with superior water-binding capacity, ultimately resulting in a denser extrudate structure with enhanced water retention properties (Dai et al., 2023).

### 3.7 Effect of lysine addition on the thermodynamic characteristics of fermented semi-dry Brown Rice noodles

From Table 2, it can be seen that fermentation significantly altered the thermodynamic characteristics of brown rice noodles. The fermentation process increased the starting temperature (To) from 47.05 °C to 57.34 °C and the peak temperature (Tp) from 49.49 °C to 61.36 °C. This may be because the enzymes and acids produced during fermentation disrupted the amorphous regions of the starch (Luo et al., 2022), promoting tighter recrystallization during noodle aging and thus increasing the gelatinization temperature. The decrease in gelatinization temperature in FBRN range also indicates enhanced homogeneity with ordered crystallites (Liang et al., 2020). However, the gelatinization enthalpy (ΔH) decreased from 0.95 J/g to 0.55 J/g, possibly due to the degradation of proteins and fats by the enzymes produced during fermentation (Zhu et al., 2019). Compared to starch-lipid/protein complexes, the enthalpy change of the structure of retrograded free starch is smaller.

**Table 2:** Effects of fermentation and lysine addition on the thermal properties of semi-dry brown rice noodles.

Samples	Onset temperature To/°C	Peak temperature Tp/°C	Endset temperature Te/°C	Gelatinization enthalpy ΔH J/g
NBRN	47.05 ± 1.41b	49.49 ± 0.08b	67.18 ± 0.23c	0.95 ± 0.02c
FBRN	57.34 ± 3.74a	61.36 ± 2.98a	67.10 ± 0.43c	0.55 ± 0.05d
FBRN-1 L	57.40 ± 0.08a	61.65 ± 0.22a	67.73 ± 1.34b	2.33 ± 0.01b
FBRN-3 L	57.43 ± 0.44a	63.14 ± 0.48a	71.53 ± 0.45b	4.14 ± 0.55a
FBRN-5 L	57.97 ± 0.21a	64.27 ± 1.34a	73.56 ± 0.04a	4.19 ± 0.04a

Note: Values are presented as mean ± standard deviation. Different letters within the same column indicate significant differences.

When lysine was added to the fermented noodles, further changes were observed. The onset, peak, and endset temperatures increased with the increase of lysine. For instance, FBRN-5 L exhibited the highest temperatures, with To at 57.97 °C, Tp at 64.27 °C, and Te at 73.56 °C. These results are similar to those reported by Liang et al., 2020, who found that the thermodynamic temperatures of corn starch increased when heated in the presence of lysine. The gelatinization enthalpy (ΔH) also rose significantly with lysine addition, particularly in FBRN-3 L (4.14 J/g) and FBRN-5 L (4.19 J/g). The rise in gelatinization temperature and enthalpy of starch observed in the DSC results after adding lysine may be due to the formation of stable complexes between lysine and starch, such as Maillard lysine-starch products (Yang et al., 1998), during the high-temperature gelatinization in rice noodle preparation. These complexes likely result in more stable structures during aging, leading to higher gelatinization temperature and enthalpy in subsequent processes.

### 3.8 Effect of lysine addition on the crystallinity of fermented semi-dry Brown Rice noodles

**Fig. 3:**
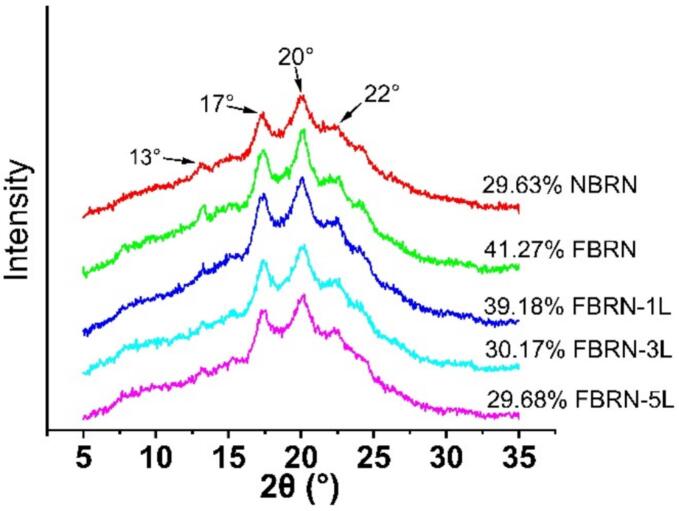
Effects of fermentation and lysine addition on the crystallinity of semi-dry brown rice noodles. (For interpretation of the references to color in this figure legend, the reader is referred to the web version of this article.)

Fig. 3 shows the X-ray diffraction (XRD) patterns of the rice noodles, with characteristic diffraction peaks at 13°, 17°, 20°, and 22°, indicating a mixture of V-type and A-type crystals. Fermentation and the addition of lysine did not change the crystalline type of the noodles. However, fermentation significantly increased the crystallinity of rice noodles to 41.27 % from 29.63 % in NBRN. This increase is primarily due to the enzymes and acids produced by lactic acid bacteria, which disrupt the amorphous regions of starch, facilitating the realignment of starch molecules into a more ordered and tightly packed crystalline structure (Luo et al., 2022). After the addition of lysine, the crystallinity of the fermented rice noodle samples decreased, dropping from 41.27 % in FBRN to 39.18 % in FBRN-1 L, 30.17 % in FBRN-3 L, and 29.68 % in FBRN-5 L. The reduction in crystallinity with lysine addition can be attributed to the formation of starch-lysine complexes, which inhibit recrystallization during the aging process of rice noodle preparation. Another possible reason is lysine interacting with starch molecules to form hydrogen bonds, thereby creating steric hindrance during the recrystallization process (Chen et al., 2021). This interaction prevents the formation of double helix structures and reduces water diffusion and exudation from starch, thereby lowering the crystallization. These findings align with the research by Ji et al., which demonstrated that lysine interactions with starch reduce overall crystallinity (Ji et al., 2016; Ji & Yu, 2018).

Although the DSC data indicated an increase in thermal stability and gelatinization enthalpy with lysine addition, the XRD results showing reduced crystallinity suggest that lysine promotes the formation of more thermally stable but less crystalline networks within the noodle matrix. This could be due to the robust lysine-starch mediated-structures, requiring higher energy to disrupt during gelatinization.

### 3.9 Effect of lysine addition on the cross-sectional morphology of fermented semi-dry Brown Rice noodles

**Fig. 4:**
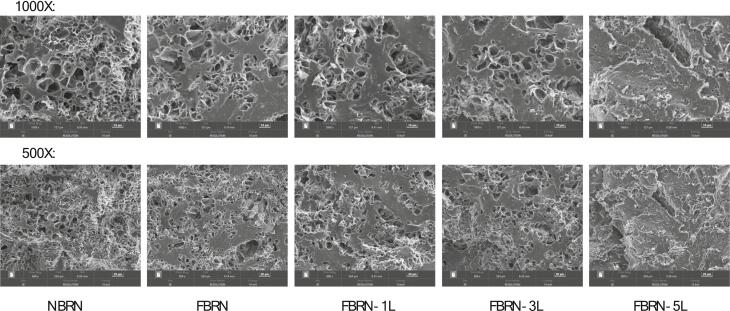
Effects of fermentation and lysine addition on the cross-sectional morphology of semi-dry brown rice noodles. (For interpretation of the references to color in this figure legend, the reader is referred to the web version of this article.)

Fig. 4 shows the scanning electron microscopy (SEM) images of the cross-sectional morphology of semi-dry rice noodles after freeze-drying. Natural brown rice noodles (NBRN) exhibited numerous pores and a loose structure, while fermented noodles (FBRN) became denser and smoother with significantly smaller and fewer voids. This change is due to fermentation degrading amorphous starch regions and increasing crystallinity, resulting in a more compact structure (as supported by DSC and XRD results). Adding 1 % and 3 % lysine had minimal impact on the FBRN structure, maintaining its dense and smooth characteristics. However, adding 5 % lysine further reduced voids, resulting in an extremely dense but rough interface structure, and the sample was brittle and prone to breakage when prepared. These SEM observations align with the results from texture profile analysis (TPA) and cooking quality assessments. Specially, FBRN-1 L and FBRN-3 L exhibit better texture and cooking properties than FBRN-5 L. This decline in FBRN-5 L's performance can be attributed to excessive lysine promoting interactions between starch molecules, leading to the formation of overly dense and compact structures. This denser structure increases the roughness of the noodle surface and brittleness, thereby deteriorating cooking quality and texture.

## 4 Conclusion

This study demonstrates that the addition of lysine and the fermentation of brown rice flour with *Lactobacillus fermentum* can significantly influence the sensory evaluation, color, pH, cooking quality, and texture of the noodles. Fermentation alone improves the texture of the noodles but introduces sourness, which can be effectively mitigated by lysine addition. Different concentrations of lysine impart distinct properties to the noodles: low to moderate lysine concentrations (1 % and 3 %) effectively reduce sourness while maintaining desirable color, texture property, and good cooking performance. Specifically, the 3 % lysine group achieved the highest sensory scores, likely due to lysine mediated starch network formation and improved water retention. However, higher lysine concentrations (5 %) caused color darkening and brittleness, compromising noodle quality. These results suggest that lysine can enhance the sensory and structural properties of fermented brown rice noodles. Future research could focus on evaluating the nutritional impact of lysine and investigating the shelf-life stability of these noodles under various storage conditions.

## CRediT authorship contribution statement

**Lijuan Luo:** Writing – original draft, Validation, Methodology, Formal analysis. **Gangping Xiong:** Writing – original draft, Methodology, Conceptualization. **Qing Wang:** Supervision, Conceptualization. **Xiongzi Xiang:** Methodology. **Zhao Long:** Methodology. **Zhengyu Huang:** Writing – review & editing. **Yuqin Ding:** Writing – review & editing, Methodology. **Chun Liu:** Writing – review & editing, Supervision, Project administration, Funding acquisition, Conceptualization.

## Declaration of competing interest

The authors declare that they have no known competing financial interests or personal relationships that could have appeared to influence the work reported in this paper.

## Data Availability

Data will be made available on request.

## Appendix A Supplementary data

Supplementary Tables S1–S4 provide additional experimental data related to the sensory evaluation, color, and water distribution of semi-dry brown rice noodles. Table S1 presents the sensory evaluation criteria for brown rice noodles, detailing the scoring system based on aroma, appearance, structure, textural properties, and taste. Table S2 summarizes the effects of fermentation and lysine addition on the sensory quality of semi-dry brown rice noodles. Table S3 shows the impact of fermentation and lysine addition on the color differences of semi-dry brown rice noodles. Table S4 describes the effects of fermentation and lysine addition on the water distribution of semi-dry brown rice noodles, including the proportions of strongly bound, weakly bound, and free water. Image 1
