# Supplementary material for: Enhancing cooking and eating quality of semi-dried brown rice noodles through Lactobacillus fermentation and moderate lysine addition
Source: Food Chem X. 2025 Mar 6;26:102327. doi: 10.1016/j.fochx.2025.102327 (PMC11930755; doi:10.1016/j.fochx.2025.102327)
Supplement: Supplementary file 1 — Supplementary Tables S1–S4 provide additional experimental data related to the sensory evaluation, color, and water distribution of semi-dry brown rice noodles. Table S1 presents the sensory evaluation criteria for brown rice noodles, detailing the scoring system based on aroma, appearance, structure, textural properties, and taste. Table S2 summarizes the effects of fermentation and lysine addition on the sensory quality of semi-dry brown rice noodles. Table S3 shows the impact of fermentation and lysine addition on the color differences of semi-dry brown rice noodles. Table S4 describes the effects of fermentation and lysine addition on the water distribution of semi-dry brown rice noodles, including the proportions of strongly bound, weakly bound, and free water. [file mmc1.docx]

**Table S1: Sensory Evaluation Criteria for Rice Noodles**

| **Primary Indicators** | **Secondary Indicators** | **Specific Characteristics** | **Score Range** |
| --- | --- | --- | --- |
| **Aroma (15 points)** | Rice aroma | Strong and rich rice aroma | 13-15 |
|  |  | Mild rice aroma | 10-12 |
|  |  | No rice aroma, but no off-flavors | 6-9 |
|  |  | No rice aroma, with off-flavors | 0-5 |
| **Appearance and Structure (25 points)** | Color | Characteristic brownish-yellow color of brown rice | 5-6 |
|  |  | Slightly darker, but no unusual colors | 3-4 |
|  |  | Bright white, yellowish, or blackish with unusual colors | 0-2 |
|  | Gloss | Obvious gloss | 4-5 |
|  |  | Slight gloss | 2-3 |
|  |  | No gloss | 0-1 |
|  | Structural integrity | Tight structure, no breakage, lumping, or powdering | 7-8 |
|  |  | Some breakage or slight lumping/powdering | 5-6 |
|  |  | Significant breakage, lumping, or powdering | 0-4 |
|  | Uniformity | Smooth surface, even thickness | 5-6 |
|  |  | Relatively smooth surface, relatively even thickness | 3-4 |
|  |  | Rough surface, uneven thickness | 0-2 |
| **Textural Properties (35 points)** | Stickiness | Smooth and moderately sticky, not adhering to teeth or undercooked | 8-10 |
|  |  | Basically non-sticky, not undercooked | 5-7 |
|  |  | Sticky or undercooked | 0-4 |
|  | Hardness | Moderately firm | 11-13 |
|  |  | Slightly soft or hard | 7-10 |
|  |  | Very soft or hard | 0-6 |
|  | Chewiness | Good chewiness | 10-12 |
|  |  | Slight chewiness | 7-9 |
|  |  | No chewiness | 0-6 |
| **Taste (25 points)** | Flavor | Strong rice flavor when chewing | 22-25 |
|  |  | Mild rice flavor when chewing | 18-21 |
|  |  | No rice flavor when chewing, no off-flavors | 15-17 |
|  |  | No rice flavor when chewing, with strong acidic or off-flavors | 0-14 |

**Table S2.** Effects of fermentation and lysine addition on the sensory quality of semi-dry brown rice noodles

| Samples | Odor | Appearance | Texture | Taste | Total score |
| --- | --- | --- | --- | --- | --- |
| NBRN | 13.1 ± 1.5a | 16.6 ± 2.5b | 22.1 ± 4.6c | 19.9 ± 1.6a | 71.7 ± 5.1a |
| FBRN | 4.6 ± 2.3d | 21.4 ± 2.1a | 26.9 ± 2.3ab | 12.3 ± 2.9b | 65.1 ± 2.9b |
| FBRN-1L | 7.6 ± 2.1c | 19.3 ± 1.0a | 27.9 ± 2.5a | 14.4 ± 2.5b | 69.1 ± 4.7ab |
| FBRN-3L | 10.0 ± 2.4b | 20.3 ± 1.7a | 24.6 ± 5.1abc | 18.4 ± 2.8a | 73.3 ± 5.4a |
| FBRN-5L | 11.6 ± 2.3ab | 16.0 ± 2.2b | 23.1 ± 3.7bc | 21.0 ± 2.1a | 71.7 ± 6.7a |

Note: Values are presented as mean ± standard deviation. Different letters within the same column indicate significant differences.

**Table S3.** Impact of fermentation and lysine addition on the color differences of semi-dry brown rice noodles

| Samples | L* | a* | b* |
| --- | --- | --- | --- |
| NBRN | 62.3±0.3b | 9.3±0.3b | 29.3±0.2c |
| FBRN | 64.5±0.2a | 8.9±0.2c | 28.5±0.3d |
| FBRN-1L | 64.6±0.1a | 8.9±0.4c | 29.2±0.2c |
| FBRN-3L | 62.0±0.1b | 9.2±0.2bc | 30.4±0.4b |
| FBRN-5L | 55.4±0.3c | 11.2±0.2a | 33.9±0.2a |

Note: Values are presented as mean ± standard deviation. Different letters within the same column indicate significant differences.

**Table S4.** Effects of fermentation and lysine addition on the water distribution of semi-dry brown rice noodles

| Samples | A21（%） | A22（%） | A23（%） |
| --- | --- | --- | --- |
| NBRN | 9.71±0.52a | 88.58±0.51bc | 1.71±0.02d |
| FBRN | 5.68±0.32d | 90.17±0.39a | 4.16±0.07b |
| FBRN-1L | 6.97±0.37c | 88.7±0.33b | 4.33±0.09a |
| FBRN-3L | 8.32±0.18b | 87.71±0.21c | 3.98±0.03c |
| FBRN-5L | 9.33±0.60a | 86.69±0.59d | 3.98±0.02c |

Note: Values are presented as mean ± standard deviation. Different letters within the same column indicate significant differences. A21, A22, and A23 represent the T21, T22, and T23 peak area percentages, indicating the content ratios of strongly bound water, weakly bound water, and free water, respectively.
